# Comparison of quinazoline and benzoylpyrazoline chemotypes targeting the CaVα-β interaction as antagonists of the N-type CaV2.2 channel

**DOI:** 10.1080/19336950.2020.1863595

**Published:** 2021-01-08

**Authors:** Dongzhi Ran, Kimberly Gomez, Aubin Moutal, Marcel Patek, Samantha Perez-Miller, Rajesh Khanna

**Affiliations:** a Department of Pharmacology, College of Medicine, The University of Arizona, Tucson, AZ, USA; b Bright Rock Path Consulting, LLC, Tucson, AZ, USA; c Comprehensive Pain and Addiction Center, The University of Arizona, Tucson, AZ, USA; d The Center for Innovation in Brain Sciences, The University of Arizona Health Sciences, Tucson, AZ, USA; e Regulonix LLC, Tucson, AZ, USA

**Keywords:** N-type/CaV2.2 antagonists, chronic pain, PPIs, CaV alpha-beta interaction

## Abstract

Structural studies with an α subunit fragment of voltage-gated calcium (CaV) channels in complex with the CaVβ subunits revealed a high homology between the various CaVα-β subunits, predicting that targeting of this interface would result in nonselective compounds. Despite this likelihood, my laboratory initiated a rational structure-based screening campaign focusing on “hot spots” on the alpha interacting domain (AID) of the CaVβ2a subunits and identified the small molecule 2-(3,5-dimethylisoxazol-4-yl)-N-((4-((3-phenylpropyl)amino)quinazolin-2-yl)methyl)acetamide (***IPPQ***) which selectively targeted the interface between the N-type calcium (CaV2.2) channel and CaVβ. ***IPPQ*** (i) specifically bound to CaVβ2a; (ii) inhibited CaVβ2 ‘s interaction with CaV.2-AID; (iii) inhibited CaV2.2 currents in sensory neurons; (iv) inhibited pre-synaptic localization of CaV2.2 *in vivo*; and (v) inhibited spinal neurotransmission, which resulted in decreased neurotransmitter release. ***IPPQ*** was anti-nociceptive in naïve rats and reversed mechanical allodynia and thermal hyperalgesia in rodent models of acute, neuropathic, and genetic pain. In structure–activity relationship (SAR) studies focused on improving binding affinity of ***IPPQ***, another compound (BTT-369), a benzoyl‐3,4‐dihydro‐1'H,2 H‐3,4'‐bipyrazole class of compounds, was reported by Chen and colleagues, based on work conducted in my laboratory beginning in 2008. BTT-369 contains tetraaryldihydrobipyrazole scaffold – a chemotype featuring phenyl groups known to be significantly metabolized, lower the systemic half-life, and increase the potential for toxicity. Furthermore, the benzoylpyrazoline skeleton in BTT-369 is patented across multiple therapeutic indications. Prior to embarking on an extensive optimization campaign of ***IPPQ***, we performed a head-to-head comparison of the two compounds. We conclude that ***IPPQ*** is superior to BTT-369 for on-target efficacy, setting the stage for SAR studies to improve on ***IPPQ*** for the development of novel pain therapeutics.

The interaction between alpha and beta subunits of the voltage-gated calcium channel alpha (CaVα/CaVβ) shapes the activity and trafficking of all subtypes of high voltage-gated calcium channels (i.e., L-, N-, P/Q-, and R-type)[1,2]. In particular, N-type voltage-gated calcium (CaV2.2) channels have been recognized as a molecular target for pain [3]. ω-conotoxin GVIA remains a defining ligand for CaV2.2 and has become the gold-standard tool for assessing CaV2.2’s role in synaptic transmission [4]. Despite intense investigation by several pharma (Xenome Ltd., Neuromed Pharmaceuticals Ltd., Zalicus Inc., Convergence Pharmaceuticals, Daiichi Sankyo), progress in clinical development of CaV2.2-targeted drugs to treat severe chronic pain has been slow notwithstanding exciting reports of efficacy in animal models (for example, TROX-1) [5]. Existing drugs targeting CaV2.2 are encumbered by toxicity (for example, intrathecal administration of the peptide Ziconotide ((Prialt®), primary alternative to morphine) [6] is associated with dizziness and sedation, while systemic administration leads to profound hemodynamic effects [7,8]). Gabapentin (Neurontin®), initially reported to target the α2δ-1-subunit of CaVα channels [9], has now been recognized to interact with NMDA-sensitive glutamate receptors, neurexin-1α, thrombospondins (adhesion molecules), and other presynaptic proteins [10], which together contribute to its adverse side effects, difficult dosing regimens, and high number needed to treat (NNT) values [11]. Mirogabalin, a gabapentinoid also targeting the α2δ-1-subunit of CaVα channels with a presumptive unique binding profile and long duration of action was discontinued in the USA and EU after the primary endpoint was not met in phase 3 trials [12]. We have advanced a strategy targeting protein interactions that modulate CaV2.2 as an alternative to direct channel blockade [13–17]. Here we report the comparison of “lead” chemotypes that emerged from two recent computational, structure-based screening efforts against the CaVα-β interaction.

In 2017, we reported the identification of the small molecule 2-(3,5-dimethylisoxazol-4-yl)-N-((4-((3-phenylpropyl)amino)quinazolin-2-yl)methyl)acetamide (***IPPQ***) targeting the CaVβ/CaVα interface [18]. This small molecule bound to CaVβ and inhibited its coupling with CaV2.2 channels, leading to a *selective* reduction in CaV2.2 currents in rat dorsal root ganglion (DRG) sensory neurons, decreased pre-synaptic localization of CaV2.2 *in vivo*, decreased frequency of spontaneous excitatory post-synaptic potentials (sEPSCs), and inhibited release of the nociceptive neurotransmitter calcitonin gene-related peptide (CGRP) from spinal cord [19]. ***IPPQ*** did not target opioid receptors nor did it engage inhibitory G protein-coupled receptor signaling. ***IPPQ*** was broadly antinociceptive in naïve animals and reversed allodynia and hyperalgesia in models of acute (post-surgical) and neuropathic (spinal nerve ligation, chemotherapy- and gp120-induced peripheral neuropathy, and genome-edited neuropathy) pain [19]. ***IPPQ*** did not cause akinesia or motor impairment, a common adverse effect of CaV2.2 targeting drugs, when injected into the brain [19]. ***IPPQ***, a 2-aminomethylquinazoline analog, represents a novel class of CaV2.2-targeting compounds that may serve as probes to interrogate CaVα-β function and ultimately be developed as a non-opioid therapeutic for chronic pain.

Developing small molecules against the CaVα/CaVβ interaction has been attempted previously. For example, prior to the crystallization of CaVβ subunits, Wyeth-Ayerst Research used high throughput yeast two-hybrid screening to identify compounds that disrupt α1b and β3 and found WAY141520, which inhibited calcium currents with an IC_50_ of 95 μM with some inhibitory activity against CaV2.2 but was not tested against other channels nor was its mechanism of action studied [20]. In 2008, my laboratory initiated a collaboration with Dr. Samy Meroueh (Indiana University) and discovered BTT-3 as an inhibitor of the CaVα/CaVβinteraction. BTT-3 could inhibit N-type currents in DRG neurons and showed anti-nociceptive activity [21]. We attempted studies to optimize this scaffold but could not improve its on-target efficacy (compounds screened up to BTT-189). After the move of my laboratory to the University of Arizona in 2014, Chen and colleagues continued this work independently and recently reported another compound (BTT-369), a benzoyl‐3,4‐dihydro‐1'H,2 H‐3,4'‐bipyrazole class of compounds, that shows marginal improvement upon BTT-3’s characteristics [22]. The compound BTT-369 identified by Chen and coworkers contains aryldihydrobipyrazole chemotype [22]; a scaffold featuring multiple phenyl groups that are known to undergo significant metabolism resulting in a lower the systemic half-life and increase potential for toxicity. Furthermore, the benzoylpyrazoline skeleton found in BTT-369 is broadly patented across multiple therapeutic indications [23,24]. Moreover, the lead compound from this study [22] was not proved to be specific for CaV2.2 and only decreased total Ca^2+^ currents at 50 µM after 48 h of incubation.

Before embarking on an extensive structure–activity relationship campaign to improve binding affinity and ADME properties of ***IPPQ***, we performed a head-to-head comparison of the two compounds. Inspection of docked poses indicated a preferred orientation of ***IPPQ*** in the AID-helix binding site of CaVβ3^25^ where the phenylpropyl group is involved in a presumed π-cation interaction with guanidinium moiety of Arg307 (Figure 1(a)). The position of the phenyl group is further stabilized by hydrophobic interactions with Val192 and Leu303. The core quinazoline scaffold reaches deep into a hydrophobic cavity contacting side chains of Met196, Leu200, A345, and making a presumed π-amide bond with Gln341. The N-H group of ***IPPQ*** amide makes a hydrogen bond with Asn340, while dimethylisozaxole is involved in multiple hydrophobic interactions with Met196, Val294, and Val299. As a result of multiple stabilizing interactions, IPPQ is nearly 75% buried in the binding cavity of the AID helical peptide and CaVα-CaVβ interface (Figure 1(a)). In contrast to ***IPPQ***, the series of recently described benzoylpyrazolines, including the lead compound BTT-369, mainly interact via the deep hydrophobic cavity (Figure 1(b)) while featuring an extra aryl group pointing out of the binding cavity toward the solvent (Figure 1(c)). The R- and S-isomers are buried only 57% and 50%, respectively. The cavity occupancy of the BTT series is thus expected to be lower compared to ***IPPQ***, which is expected to result in a lower ligand affinity due to multiple sampling of the binding cavity. Furthermore, ***IPPQ*** shows a better overlap with AID helix residues known to be key for binding in this pocket [25,26] (Figure 1(d)).

**Figure 1.**
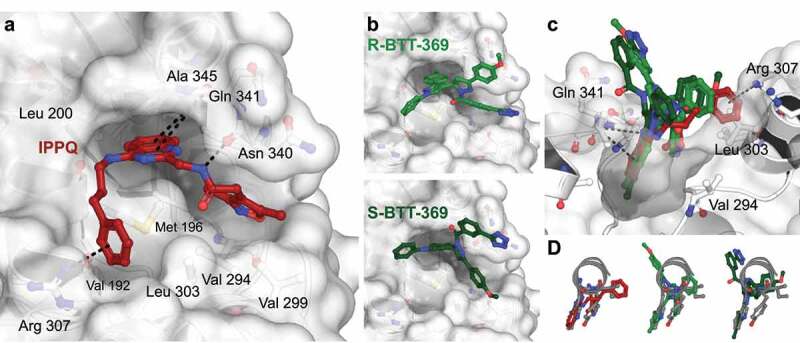
A. Docking analysis of IPPQ and BTT-369 binding to CaVβ highlights differences in cavity occupancy. (a) IPPQ and (b) BTT-369 docked to the AID-binding cavity of the rat CaVβ3 subunit (PDB ID 1vyt [25]). (c) Rotated view shows that both isomers of BTT-369 project from the binding pocket. (d) Overlap with conserved residues Tyr467, Trp470, and Ile471 from co-crystallized rat CaVα1 c (1.2) AID helix. Docking scores: −7.0 for IPPQ, −6.3 for R-BTT-369, −6.1 for S-BTT-369

Beyond in silico analysis of the lead compounds from the ***IPPQ*** [19] or the BTT [22] series an important outcome of inhibiting the CaVα-CaVβ interaction is the control of the peregrination of the calcium channel to the plasma membrane [2]. In the original study identifying ***IPPQ***, CaV2.2 membrane localization was never investigated. Similarly, while the non-cell permeant analog BTT-266 was able to decrease CaV2.2 membrane localization in heterologous cells, BTT-369’s activity remained uncharacterized [22]. Therefore, we tested if ***IPPQ*** and BTT-369 could decrease CaV2.2 membrane localization in rat DRG neurons. BTT-369 was obtained from TCG Life Sciences as a racemic mixture from which the R- and S-enantiomers were separated by chromatography on a chiral column. As a consequence, the absolute stereochemistry of each fraction was not determined, and therefore we will identify the respective enantiomers as BTT-369A and BTT-369B for the remainder of this study. Primary cultures of adult rat sensory neurons were made and treated overnight with 20 µM of either ***IPPQ***, BTT-369 (racemic mixture, R- or S-enantiomers) or vehicle (0.1% DMSO). Neurons were fixed on the next day and stained for CaV2.2 (Figure 2(a)). Analysis using confocal microscopy showed that in vehicle treated neurons, CaV2.2 was localized at the plasma membrane (surface CaV2.2 index: 0.93 ± 0.054, n = 13) as expected (Figure 2(a)). In ***IPPQ***-treated sensory neurons, CaV2.2 membrane localization was decreased (surface CaV2.2 index: 0.49 ± 0.047, p < 0.0001, n = 16). In contrast, racemic BTT-369 (surface CaV2.2 index: 0.95 ± 0.05, p > 0.9999, n = 19), BTT-369A (surface CaV2.2 index: 0.78 ± 0.056, p = 0.1811, n = 15) and BTT-369B (surface CaV2.2 index: 0.80 ± 0.037, p = 0.4966, n = 20) had no significant effect on CaV2.2 membrane localization (Figure 2(a and b)). These results demonstrate that the quinazoline derivate ***IPPQ*** can efficiently control the trafficking of CaV2.2 leading to decreased membrane localization of the channel in rat DRG neurons. We cannot rule out that beyond inhibition of the alpha–beta interaction, that ***IPPQ*** may also act on free beta subunits. The benzoylpyrazoline derivate BTT-369 had no effect on CaV2.2 trafficking in either racemic, R- or S-form. Thus, the treatment of sensory neurons with 20 µM of ***IPPQ*** replicated the well-described effect of the loss of CaV2.2-CaVβ interaction on the trafficking of the channel [2]. BTT-369’s failure to decrease CaV2.2 membrane localization may argue against an action directly via CaVβ subunits.

**Figure 2.**
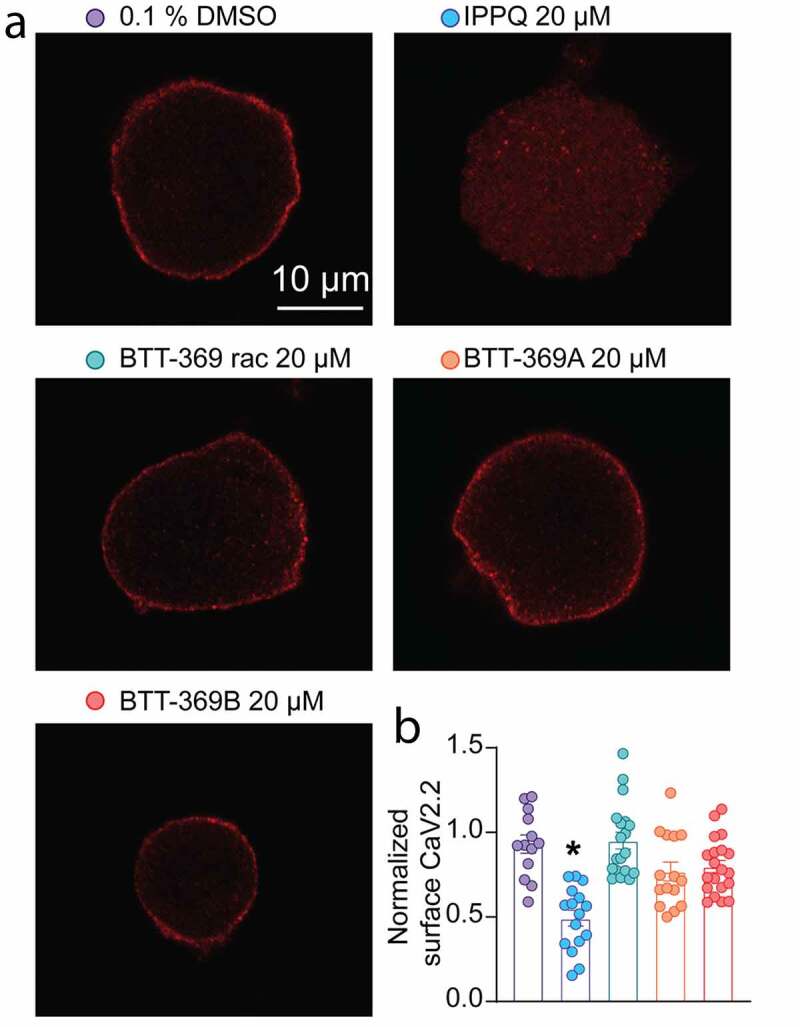
Modulation of CaV2.2 membrane localization in rat DRG neurons by the CaVβ interfering compounds ***IPPQ*** or BTT-369. (a) Representative micrographs of CaV2.2 staining in rat sensory neurons treated with either IPPQ, BTT-369 (racemic mixture, R- or S- enantiomers) or vehicle (0.1% DMSO) overnight. Scale bar is 10 µm. (b) Bar graph with scatter plot of the normalized surface expression of NaV1.7 per sensory neuron (n = 13 to 20 cells per condition). IPPQ decreased CaV2.2 surface localization while BTT-369 had no effect in either combination of R-, S- and racemic mixture. *p < 0.05, Kruskal-Wallis test with Dunn’s multiple comparison post-hoc test. Error bars indicate mean ± SEM

The CaVβ subunits are essential for the proper function of calcium channels [2]. In small diameter sensory neurons, CaVβ3 expression facilitates high-voltage gated Ca^2+^ channel function [27]. We previously validated the specificity of ***IPPQ*** for CaV2.2 in DRG neurons [19]. In contrast, while the non-cell permeant analog BTT-266 was evaluated in rat DRG neurons, the lead compound of the benzoylpyrazoline series BTT-369 was only tested in heterologous cells [22]. Therefore, we tested the ability of the CaVβ interfering compounds ***IPPQ*** or BTT-369 to inhibit Ca^2+^ currents in DRG neurons. We made primary adult rat DRG neurons and treated them overnight with 20 µM of either ***IPPQ***, BTT-369 (racemic mixture, R- or S-enantiomers) or vehicle (0.1% DMSO). We isolated N-type Ca^2+^ current using a combination of toxins and small molecules to block the other calcium channel subtypes (Figure 3(a)). As before [19], ***IPPQ*** inhibited CaV2.2 currents by ~57% (DMSO: −48.47 ± 4.5 pA/pF vs *IPPQ*: −20.90 ± 3.2 pA/pF, p = 0.0002, n = 14 and 15 cells, respectively) (Figure 3(b and c)). Racemic BTT-369 (−39.51 ± 6.1 pA/pF, p > 0.9999, n = 13), BTT-369A (−39.81 ± 6.0 pA/pF, p > 0.9999, n = 14) and BTT-369B (−31.66 ± 3.7 pA/pF, p = 0.1114, n = 18) had no significant effect on CaV2.2 currents (Figure 3(b and c)). This shows that although BTT-369 can decrease CaV2.2 currents in heterologous cells, this does not hold true in the cell of interest for pain, i.e. DRG neurons. ***IPPQ*** did not affect the V½ of activation but caused a ~ 6-mV shift in the V½ of inactivation and a slight increase in the slope (*k*) of activation of CaV2.2 channels (Figure 3(d and e); Table 1). BTT-369A also shifted the V½ of activation by ~4-mV while both BTT-369 racemic and BTT-269B accelerated the slope of activation; inactivation *k* was also increased by both BTT-369 racemic and BTT-269B (Table 1).

**Table 1.** Gating properties of N-type calcium currents from DRG neurons treated with N-type antagonists

	DMSO	***IPPQ***	BTT-369 racemic	BTT-369A	BTT-369B
	0.1%		20 µM		
Activation					
*V_1/2_*	2.3 ± 0.7(11)	-3.9 ± 1.5(14)	3.6 ± 1.0(13)	6.7 ± 0.8(12)*^a^*	5.9 ± 1.1(16)
*k*	5.0 ± 0.6(11)	7.3 ± 1.2(14)*^b^*	6.1 ± 0.8(13)*^c^*	5.6 ± 0.7(12)	6.7 ± 0.9(16)*^d^*
Inactivation					
*V_1/2_*	-14.9 ± 2.2(15)	-20.9 ± 2.2(15)*^e^*	-11.7 ± 6.0(12)	-10.3 ± 2.0(12)*^f^*	-13.7 ± 3.1(16)
*k*	-12.1 ± 1.7(15)	-13.6 ± 1.9 (15)	-15.9 ± 3.9 (12)*^g^*	-12.0 ± 1.3(12)	-14.5 ± 2.2(16)*^h^*

Values are means ± S.E.M. calculated from fits of the data from the indicated number of individual DRG neurons (in parentheses) from female rats to the Boltzmann equation; *V_1/2_* midpoint potential (mV) for voltage-dependent activation or inactivation; *k*, slope factor. These values pertain to Figure 3. *^a^*p = 0.0408, *^b^*p < 0.0001, *^c^*p = 0.0157, *^d^*p < 0.0001; *^e^*p < 0.0001, *^f^*p = 0.0026; *^g^*p = 0.0003, *^h^*p = 0.0181 vs DMSO (one-way ANOVA with Dunnett’s post-hoc test).

**Figure 3.**
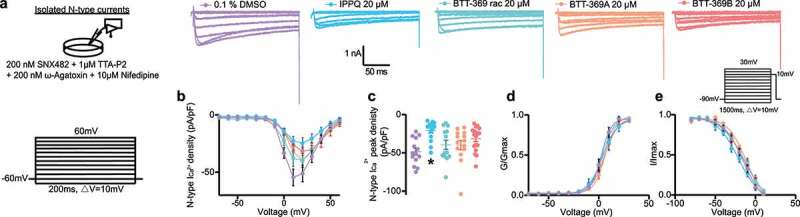
Inhibition of CaV2.2 currents by the CaVβ interfering compounds ***IPPQ*** or BTT-369. (a) Representative calcium current (via N-type channels) traces recorded from small-sized rat DRG neurons, incubated overnight with the indicated compounds, in response to holding voltage of −60 mV with 200-ms voltage steps applied at 5-s intervals in +10 mV increments from −70 to +60 mV. Pharmacological isolation of N-type (CaV2.2) current was achieved with a cocktail of toxins/small molecules. (b) Summary of current-voltage curves and (c) normalized peak currents (pA/pF) from DRG neurons as indicated. Boltzmann fits for normalized conductance G/Gmax voltage relations for voltage-dependent activation (d) and inactivation I/Imax (e) of the sensory neurons as indicated. Error bars indicate mean ± s.e.m. Half-maximal activation and inactivation (V1/2) and slope values (k) for activation and inactivation were not different between any of the conditions (p > 0.9999, Kruskal-Wallis test with Dunn’s post hoc); values presented in Table 1. *p < 0.05, Kruskal-Wallis test with Dunn’s multiple comparison post-hoc test. Error bars indicate mean ± SEM

While the initial hit ***IPPQ*** originated from commercial sources, a 2-aminomethylquinazoline scaffold has been underexplored as a lead-like scaffold. Indeed, a Tier 1 patent search confirmed a favorable IP position with no patent or literature references on the ***IPPQ*** chemotype. When surveying the ***IPPQ*** chemotype for an off-target activity, we have noticed the kinase hinge-binding motif of 4-aminoquinazolines. While the cross-target selectivity will be assessed during lead optimization, CHEMBL and PubMed search revealed no targets that would interact with ***IPPQ***. We seek to develop ***IPPQ*** analogs that do not cross the blood:brain barrier (BBB) or have limited CNS penetration, thus limiting neurologic/psychiatric adverse effects. Due to the presence of polar residues and two polar hydrogen bond donors, we do not expect ***IPPQ*** to cross the BBB to a significant extent.

## Methods

### Ethics statement regarding use of animals

All animal use was conducted in accordance with the National Institutes of Health guidelines, and the study was carried out in strict accordance with recommendations in the Guide for the Care and Use of Laboratory Animals of the University of Arizona (Protocol #: 16–141). Pathogen-free adult female Sprague-Dawley rats (125 g; Envigo, Indianapolis, IN) were housed in temperature-controlled (23 ± 3 °C) and light-controlled (12-h light/12-h dark cycle; lights on 07:00–19:00) rooms with standard rodent chow and water available ad libitum.

### Preparation of acutely dissociated dorsal root ganglion neurons

Dorsal root ganglia (DRG) from all levels were acutely dissociated using methods as described previously (17, 19, 28). DRG neurons were isolated from ~100 g Sprague-Dawley female rats. DRG were then collected, trimmed at their roots, and enzymatically digested in 3 mL bicarbonate-free, serum-free, sterile DMEM (Cat# 11,965, Thermo Fisher Scientific, Waltham, MA) solution containing neutral protease (3.125 mg.ml-1, Cat#LS02104; Worthington, Lakewood, NJ) and collagenase type I (5 mg/mL, Cat# LS004194, Worthington, Lakewood, NJ) and incubated for 60 minutes at 37°C under gentile agitation. Dissociated DRG neurons were then gently centrifuged to collect cells and resuspended with DRG media DMEM containing 1% penicillin/streptomycin sulfate, 30 ng/mL nerve growth factor, and 10% fetal bovine serum (Hyclone) before plating onto poly-D-lysine – and laminin-coated glass 12-mm coverslips. Thirty minutes after, DMSO (0.1%), ***IPPQ*** (20 µM), and BTT-369 compounds (20 or 50 µM) were added into the cells overnight.

### Whole-cell patch clamp recordings of N-type (CaV2.2) Ca2+ currents in acutely dissociated DRG neurons

To isolate the N-type currents in DRG, contributions of the high- and low-voltage-activated calcium channel subtypes, as well as sodium currents, were blocked with the following selective blockers (all purchased from Alomone Labs, Jerusalem): Nifedipine (10 µM, L-type); ω-agatoxin GIVA (200 nM, P/Q-type); SNX-482 (200 nM, R-type); TTA-P2 (1 µM, T-type) and TTX (1 µM, Na^+^ channels). Extracellular recording solution (at ~310 mOsm) consisted of the following (in mM): 110 N-methyl-D-glucamine, 10 BaCl_2_, 30 tetraethylammonium chloride, 10 HEPES, 10 D-glucose, pH adjusted to 7.4. The intracellular recording solution (at ~310 mOsm) consisted of the following (in millimolar): 150 CsCl_2_, 10 HEPES, 5 Mg-ATP, 5 BAPTA, pH adjusted to 7.2. Activation of N-type currents was measured using a holding voltage of −60 mV with 200-ms voltage steps applied at 5-s intervals in +10 mV increments from −70 to +60 mV. Current density was calculated as peak I_Ca_ divided by cellular capacitance. Steady-state inactivation of N-type currents was determined by applying a 1500 ms conditioning prepulse (−100 to +30 mV in +10 mV increments) after which, the voltage was stepped to +10 mV for 200-ms; a 15-s interval separated each acquisition.

Fire-polished recording pipettes, 2 to 4 MΩ resistance, were used for all recordings. Whole-cell recordings were obtained with a HEKA EPC-10 USB (HEKA Instruments Inc., Bellmore, NY); data were acquired with a Patchmaster (HEKA) and analyzed with a Fitmaster (HEKA). Capacitive artifacts were fully compensated, and series resistance was compensated by ~70%. Recordings made from cells with greater than a 20% shift in series resistance compensation error were excluded from analysis. All experiments were performed at room temperature (~23°C).

### Immunocytofluorescence, confocal microscopy, and quantification of surface CaV2.2

Immunocytofluorescence was performed on cultured primary adult rat DRGs as described previously [28]. Briefly, cells were fixed using ice-cold methanol for 5 minutes and allowed to dry at room temperature. Cells were rehydrated in PBS and anti-CaV2.2 antibody (Cat#TA308673, Origene, Rockville, MD, 1/1000) was added in PBS with 3% BSA for 1 h at room temperature. Cells were then washed three times in PBS and incubated with PBS containing 3% BSA and secondary antibodies (Alexa 594 goat anti-rabbit from Thermofisher) for 1 h at room temperature. After washing with PBS, cells were stained with 4',6-diamidino-2-phenylindole (DAPI, 50 µg/ml) and mounted in Fluoro-gel (Cat# 17985, electron microscopy sciences, Hatfield, PA). Immunofluorescent micrographs were acquired using a plan-Apochromat 63x/0.8 objective on a Zeiss LSM880 confocal microscope operated by the Zen Black software (Zeiss). Camera gain and other relevant settings were kept constant. Membrane immunoreactivity was calculated by measuring the signal intensity in the area contiguous to the boundary of the cell. Membrane to cytosol ratio was determined by defining regions of interest on the cytosol and the membrane of each cell using Image J. Total fluorescence was normalized to the area analyzed and before calculating the ratios.

### Computational approaches

Preparation and docking were conducted using Schrödinger Release 2019–3 (Schrödinger, LLC, New York, NY, 2020) [29]. IPPQ, S- and R-BTT-369 were prepared using LigPrep with possible ionization states at pH 7.0. The structure of rat CaVβ3 co-crystallized with the AID domain of rat CaVα1 c (PDB ID 1yvt [25]) was prepared using the Protein Preparation Wizard [30]. A 20x20x20 Å box was centered on the AID helix to generate a grid suitable for peptide docking. Docking was performed with Glide Standard Precision (SP)-peptide mode. The best poses for each compound were selected based both on visual inspection and docking scores. Buried surface areas were calculated using Areaimol [31]. Figures were generated with the PyMOL Molecular Graphics System, Version 2.0 Schrödinger, LLC.

### Statistical analyses

All data were first tested for a Gaussian distribution using a D’Agostino-Pearson test (Prism 9 Software, Graphpad, San Diego, CA). All data were analyzed using the non-parametric Kruskal–Wallis test followed by Dunn’s post-hoc test. Differences were considered significant if p ≤ 0.05. Error bars in the graphs represent mean ± SEM. All data were plotted in Prism 9.

## Data Availability

Please contact author for data requests.
